# Clinical Applications and Diagnostic Performance of Adjunctive Light-Based Optical Technologies in Oral Potentially Malignant Disorders and Squamous Cell Carcinoma: A Systematic Review

**DOI:** 10.3390/jcm15051693

**Published:** 2026-02-24

**Authors:** Dariusz Paliga, Magdalena Kronenberg, Małgorzata Pihut, Magdalena Pietrzko, Dariusz Skaba, Rafał Wiench

**Affiliations:** 1 Dental Practice Renata and Dariusz Paliga S.C., Al. Niepodleglosci 3 lok 2, 35-303 Rzeszow, Poland; 2 Student Research Group, Chair and Department of Pathomorphology, Faculty of Medical Sciences in Zabrze, Medical University of Silesia, 40-055 Katowice, Poland; patolog@interia.pl; 3 Prosthodontic and Orthodontic Department, Jagiellonian University Medical College, 4 Montelupich Str., 31-155 Krakow, Poland; malgorzata.pihut@uj.edu.pl; 4 Privat Dental Office Stomatologia Pietrzko, Budowlanych Str. 1, 43-300 Bielsko-Biała, Poland; pietrzkoperio@gmail.com; 5 Department of Periodontal Diseases and Oral Mucosa Diseases, Faculty of Medical Sciences in Zabrze, Medical University of Silesia, 40-055 Katowice, Poland; rwiench@sum.edu.pl

**Keywords:** oral cancer screening, oral potentially malignant disorders, squamous cell carcinoma, autofluorescence, chemiluminescence, optical imaging, diagnostic accuracy

## Abstract

**Background:** Oral squamous cell carcinoma often develops from oral potentially malignant disorders and is frequently diagnosed at an advanced stage. Conventional oral examination is limited by moderate sensitivity, observer variability, and poor discrimination between benign and dysplastic lesions. Adjunctive light-based screening technologies have been introduced, but their diagnostic value remains uncertain. **Methods:** This systematic review followed PRISMA 2020 guidelines and was registered in PROSPERO. MEDLINE (PubMed), Embase, Scopus, and the Cochrane Database were searched through December 2025. Studies assessing adjunctive light-based screening technologies for detecting oral potentially malignant disorders or squamous cell carcinoma were included. Histopathology served as the reference standard. Diagnostic accuracy outcomes were extracted, and risk of bias was assessed using Cochrane-based criteria. **Results:** Eleven studies were included. Autofluorescence imaging showed consistently high sensitivity but low and variable specificity. Chemiluminescence demonstrated similar or lower sensitivity with poor specificity. False-positive results were frequent, particularly in inflammatory or benign lesions. Marked heterogeneity across studies limited quantitative synthesis. **Conclusions:** Adjunctive light-based technologies can increase detection sensitivity when used with conventional oral examination but lack sufficient specificity for standalone use. Histopathological confirmation remains mandatory. Standardized, multicenter diagnostic accuracy studies are needed to clarify their clinical role.

## 1. Introduction

### 1.1. Rationale

Oral cancers represent a substantial global public health challenge, with 758,000 new cases diagnosed worldwide in 2022, accounting for approximately 7% of all cancers globally [1,2]. Squamous cell carcinoma comprises over 90% of these malignancies, with oral cavity cancer representing approximately 42% of cases, followed by oropharyngeal cancer at 19.3% [2]. Despite advances in treatment modalities, the 5-year survival rate for advanced oral cancer remains approximately 25–60%, largely due to late-stage presentation [3]. In contrast, early-stage disease treated with surgery or radiotherapy achieves 5-year survival rates of 70–90% [3]. This stark disparity underscores the critical importance of early detection strategies. A significant proportion of oral squamous cell carcinomas arise from oral potentially malignant disorders (OPMDs), including leukoplakia, erythroplakia, oral submucous fibrosis, and proliferative verrucous leukoplakia [4,5]. The malignant transformation rates of these lesions vary considerably: oral leukoplakia demonstrates transformation rates of 8.4–9.5%, oral erythroplakia shows rates as high as 33–50%, and proliferative verrucous leukoplakia exhibits the highest risk at approximately 49.5% [6,7,8]. The median time to malignant transformation for oral leukoplakia is approximately 25 months, emphasizing the window of opportunity for intervention [7]. Recognition and appropriate management of OPMDs therefore represents a crucial strategy for oral cancer prevention and early detection. Conventional oral examination (COE), consisting of systematic visual inspection and palpation of the oral cavity and oropharynx, remains the primary screening method for detecting oral cancer and OPMDs [9,10]. However, COE has significant limitations. Studies demonstrate that COE achieves a sensitivity of only 63–71% and specificity of 78–85% for detecting dysplastic and malignant lesions in patients presenting with clinically evident abnormalities [3,11]. In screening asymptomatic populations, sensitivity estimates range widely from 50% to 99%, reflecting substantial variability in examiner expertise and lesion characteristics [10]. Furthermore, COE cannot reliably distinguish between benign inflammatory conditions and dysplastic lesions, nor can it accurately delineate lesion margins for biopsy guidance [12,13]. The subjective nature of visual assessment, coupled with the subtle clinical appearance of early dysplastic changes, contributes to delayed diagnosis and missed opportunities for early intervention. To address these limitations, adjunctive light-based screening technologies have been developed to enhance the detection and characterization of oral mucosal abnormalities. These technologies exploit the optical properties of normal and abnormal oral tissues, including differences in autofluorescence patterns, light absorption, and scattering characteristics [14]. The two most widely studied light-based adjuncts are autofluorescence imaging and chemiluminescence-enhanced visualization. Autofluorescence devices, such as VELscope and OralID, utilize blue light excitation (typically 400–460 nm) to induce fluorescence from endogenous fluorophores including flavin adenine dinucleotide (FAD), collagen, and porphyrins [14]. Dysplastic and malignant tissues exhibit altered fluorescence patterns, appearing as areas of fluorescence visualization loss (FVL) due to changes in tissue architecture, increased cellular metabolism, and altered stromal composition [15,16]. Chemiluminescence systems, such as ViziLite and Microlux/DL, employ diffuse white light following acetic acid rinse to enhance visualization of abnormal epithelium, which appears acetowhite due to increased nuclear density and altered protein content [17,18]. Previous systematic reviews and meta-analyses have reported variable diagnostic accuracy for these technologies. For autofluorescence, pooled sensitivity estimates range from 81–90% with specificity of 50–72% [19,20,21]. Chemiluminescence demonstrates sensitivity of 67–85% but lower specificity of 48–52% [19,20]. However, these studies have been limited by heterogeneity in study design.

### 1.2. Objectives

The primary objective of this systematic review was to evaluate the diagnostic accuracy of adjunctive light-based screening technologies, including autofluorescence, chemiluminescence, and other optical visualization methods, for the detection of oral potentially malignant disorders and squamous cell carcinoma. The secondary objectives were to assess false-positive and false-negative rates, interobserver agreement where reported, and factors influencing diagnostic performance, including lesion type, anatomical location, and clinical setting. In addition, this review aimed to identify methodological limitations within the existing literature and to clarify the potential clinical role of light-based adjuncts in oral cancer screening and surveillance.

Given the diversity of clinical applications of light-based optical technologies in oral oncology, studies were categorized a priori into two clinical domains. In view of the expanding clinical use of light-based optical technologies across different stages of oral oncologic management, this review was designed to address two distinct but related clinical domains: (1) diagnostic assessment and triage of oral mucosal lesions for biopsy, and (2) intraoperative optical guidance during surgical management of oral squamous cell carcinoma. These domains represent different points along the clinical pathway and were synthesized separately to avoid conflation of diagnostic accuracy outcomes with surgical performance endpoints.

## 2. Materials and Methods

### 2.1. Focused Question

This review incorporated two predefined clinical questions structured according to the PICO framework.

- Diagnostic Assessment: In adult patients presenting with oral mucosal lesions or potentially malignant disorders (Population), what is the diagnostic performance of adjunctive light-based visualization technologies, including autofluorescence and chemiluminescence (Index test), compared with histopathological examination (Reference standard), for the detection of epithelial dysplasia or squamous cell carcinoma (Outcome)?
- Intraoperative Guidance: In adult patients undergoing surgical treatment for oral squamous cell carcinoma (Population), does the use of fluorescence- or light-guided surgical techniques (Intervention), compared with conventional surgery or surgeon visual assessment (Comparator), improve surgical or oncologic outcomes such as margin status, lymph node yield, or recurrence (Outcomes)?” [22].

### 2.2. Search Strategy

This systematic review was registered in the PROSPERO database (registration number: CRD420261279754) and conducted in accordance with the PRISMA 2020 guidelines (Supplementary Materials S1) [23]. A comprehensive electronic literature search was performed in MEDLINE (via PubMed), Embase, Scopus, and the Cochrane Database of Systematic Reviews, covering studies published up to 22 December 2025. The search was carried out in December 2025. The search strategy, (“oral screening device*” [Title/Abstract] OR autofluorescence [Title/Abstract] OR “tissue autofluorescence” [Title/Abstract] OR chemiluminescence [Title/Abstract] OR “optical imaging” [Title/Abstract] OR “light-based examination” [Title/Abstract] OR VELscope [Title/Abstract] OR Vizilite [Title/Abstract] OR “fluorescence visualization” [Title/Abstract]) AND (“oral dysplasia” [Title/Abstract] OR “oral epithelial dysplasia” [Title/Abstract] OR “oral potentially malignant disorder*” [Title/Abstract] OR “oral premalignant lesion*” [Title/Abstract] OR leukoplakia [Title/Abstract] OR erythroplakia [Title/Abstract] OR “oral squamous cell carcinoma” [Title/Abstract] OR “oropharyngeal dysplasia” [Title/Abstract] OR “oropharyngeal cancer” [Title/Abstract]), incorporated terms related to adjunctive light-based diagnostic and screening technologies for oral lesions, including autofluorescence, chemiluminescence, optical imaging, and visually enhanced lesion visualization devices, as well as terms describing oral potentially malignant disorders and squamous cell carcinoma. Therapeutic laser modalities were explicitly excluded from the search strategy. In addition, a manual snowball search was conducted by screening the reference lists of all articles included for full-text review to identify potentially relevant studies not captured in the electronic search. Google Scholar was used to verify citation completeness and identify additional eligible records. The search was restricted to studies published in English in accordance with the predefined inclusion criteria. All databases were independently searched by three reviewers, using identical search strategies. Study eligibility was assessed collaboratively by all authors during the screening process. Data extraction from the included studies was performed independently by two reviewers, with discrepancies resolved through discussion and consensus [24].

### 2.3. Outcome Measures

The primary outcomes of this systematic review were diagnostic accuracy parameters of adjunctive light-based screening technologies for oral lesions. These included sensitivity, specificity, positive predictive value, negative predictive value, and overall diagnostic accuracy for the detection of epithelial dysplasia and squamous cell carcinoma. Where available, likelihood ratios and area under the receiver operating characteristic curve were also extracted. Histopathological examination of biopsy specimens served as the reference standard. In studies where histology was not universally performed, expert clinical diagnosis with mandatory biopsy of suspicious lesions was accepted as a secondary reference standard and analyzed separately. Secondary outcomes included false-positive and false-negative rates, interobserver agreement, diagnostic performance stratified by lesion type or anatomical location, and reported limitations or adverse consequences related to the use of adjunctive screening devices, such as unnecessary biopsies or patient anxiety. Time required for examination and feasibility in routine clinical practice were recorded when reported. Outcomes were analyzed separately according to the predefined clinical domain. For diagnostic assessment studies, primary outcomes included sensitivity, specificity, predictive values, and overall diagnostic accuracy. For intraoperative guidance studies, outcomes included margin concordance with final histopathology, lymph node yield, recurrence rates, and other surgical performance indicators. Quantitative pooling across domains was not performed due to fundamental differences in study design and outcome definitions.

Due to heterogeneity in study design, index tests, thresholds for positivity, clinical settings, and outcome definitions, a quantitative meta-analysis was not performed. Instead, studies were grouped according to predefined clinical domains and further stratified within the diagnostic assessment domain by:

(1) device modality (autofluorescence, chemiluminescence, multimodal imaging),
(2) clinical setting (screening/general dental vs. specialist referral),
(3) lesion spectrum (OPMD-predominant vs. OSCC-enriched cohorts),
(4) positivity threshold definition.

### 2.4. Selection of Studies

This systematic review aimed to evaluate the diagnostic accuracy of adjunctive light-based screening technologies, including autofluorescence, chemiluminescence, and other optical visualization methods, for the detection of oral potentially malignant disorders and squamous cell carcinoma. Studies were eligible for inclusion if they assessed one or more adjunctive light-based screening devices used for diagnostic or screening purposes and reported outcomes related to diagnostic accuracy, with histopathological confirmation as the reference standard. Both prospective and retrospective clinical studies were considered. Narrative reviews, animal studies, in vitro studies, and studies focused exclusively on therapeutic laser applications were excluded. The detailed inclusion and exclusion criteria are presented in Table 1.

### 2.5. Quality Assessment and Risk of Bias Across Studies

The methodological quality and risk of bias of the included studies were independently assessed by two reviewers using a predefined, custom nine-item assessment tool adapted from Cochrane methodological principles [25]. The tool evaluated key domains relevant to diagnostic and clinical studies, including clarity of the study protocol, adequacy of diagnostic confirmation, appropriateness of control or comparison groups, description of randomization where applicable, suitability of statistical analysis, transparency of outcome reporting, completeness of follow-up and attrition reporting, and disclosure of funding sources and conflicts of interest. Each item was judged as fulfilled, not fulfilled, or unclear. Overall risk of bias was categorized as low, moderate, or high based on the collective pattern of item-level judgments rather than strict numerical thresholds. Any discrepancies between reviewers were resolved through discussion and consensus.

### 2.6. Data Extraction

Following final study inclusion, two reviewers independently extracted data using a predefined and standardized data extraction form. The following information was collected:

- Study characteristics: first author, year of publication, country, and study design.
- Participants: sample size, age range or mean age, and sex distribution.
- Index test: type of adjunctive light-based screening technology used and examination protocol.
- Reference standard: histopathological confirmation method and biopsy criteria.
- Diagnostic accuracy outcomes: sensitivity, specificity, positive predictive value, negative predictive value, likelihood ratios, and overall diagnostic accuracy, where reported.
- Additional outcomes: false-positive and false-negative rates, interobserver agreement, and reported clinical limitations or adverse consequences of screening.
- Follow-up: interval between index test and reference standard, when applicable.

Any disagreements during data extraction were resolved through discussion to ensure data accuracy and consistency. Diagnostic accuracy parameters were extracted as reported in the original studies. Where studies presented data per lesion, each lesion was considered the unit of analysis. Where data were reported per patient, the patient constituted the analytical unit. No attempt was made to convert lesion-level data to patient-level estimates due to insufficient reporting of clustering parameters.

## 3. Results

### 3.1. Study Selection

**Figure 1:**
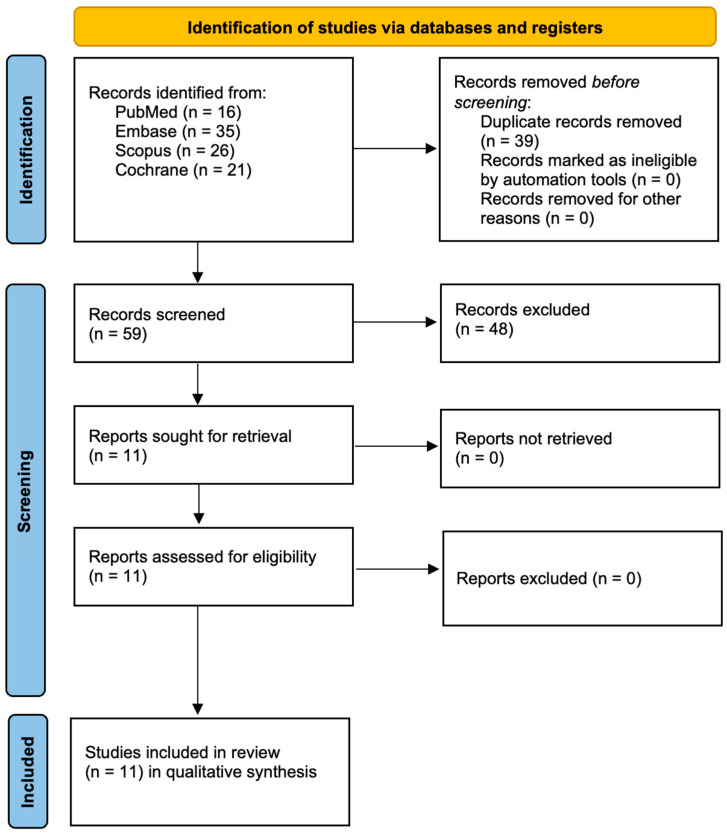
Prisma 2020 flow diagram.

The database search identified a total of 98 records, including 16 from PubMed, 35 from Embase, 26 from Scopus, and 21 from Cochrane. After removal of 39 duplicate records, 59 records remained and were subjected to title and abstract screening. During this stage, 48 records were excluded. The full texts of the remaining 11 reports were sought and successfully retrieved. All 11 reports were subsequently assessed for eligibility, with no exclusions at this stage. Ultimately, 11 studies were included in the qualitative synthesis. This is shown in Figure 1.

### 3.2. Risk of Bias and Quality Assessment of Evidence Results

The risk of bias for each study was evaluated in a structured manner, and an overall judgment of low, moderate, or high risk was assigned in line with the Cochrane Handbook for Systematic Reviews of Interventions [24]. None of the 11 included studies were classified as having a high risk of bias. Six studies obtained a total score of eight points, while two achieved seven points. Although no study reached the maximum score of nine, all were retained, as the missing or unclear items were considered unlikely to compromise the validity of the overall evidence appraisal. Each adequately met criterion contributed one point, whereas criteria that were unmet, unclear, or ambiguously reported were not scored. The individual criteria and corresponding study scores are detailed in Table 2.

### 3.3. General Characteristics of the Included Studies

The included studies were categorized according to the predefined clinical domains. Studies evaluating chairside diagnostic assessment of mucosal lesions were analyzed separately from studies assessing intraoperative fluorescence-guided surgical techniques. Outcomes from these domains are presented independently. A total of 11 studies published between 2015 and 2025 were included in the qualitative synthesis. The studies originated from diverse geographical regions, including Europe, the Middle East, and Asia. Considerable heterogeneity was observed in study design, encompassing prospective clinical studies, randomized controlled trials, randomized clinical trials, single-arm intervention studies, and non-randomized clinical investigations. Only a limited number of studies reported a formal a priori sample size calculation, typically based on statistical power analysis or predefined allocation methods. Sample sizes varied substantially across the included studies, reflecting differences in methodological design and clinical setting. The majority of studies employed parallel-group designs. Demographic reporting was inconsistent across studies. Where reported, participant age spanned a wide range from early adulthood to older age, indicating clinically heterogeneous populations. Sex distribution varied between studies, with some reporting a predominance of female participants and others demonstrating a more balanced male-to-female ratio. No study was excluded due to incomplete demographic reporting. Table 3 provides a general overview of the studies.

The included studies demonstrated heterogeneous designs and outcomes across different clinical contexts and light-based technologies. In a randomized clinical trial from Denmark, Christensen et al. showed that near-infrared fluorescence–guided neck dissection significantly increased lymph node yield compared with conventional surgery, particularly in cervical levels Ib–III [26]. A study by Cicciù et al. described fluorescence imaging as a non-invasive adjunct for early detection of oral premalignant and malignant lesions, although no quantitative diagnostic accuracy outcomes were reported [27]. In a multicenter randomized clinical trial from Canada, Durham et al. found that fluorescence visualization–guided surgery did not reduce 3-year local recurrence rates and did not improve margin status, survival, or recurrence compared with conventional surgery [28]. A prospective Japanese study by Ikeda et al. demonstrated that fluorescence visualization delineated oral epithelial dysplasia adjacent to early tongue squamous cell carcinoma comparably to iodine vital staining, with fluorescence visualization loss correlating with increased expression of CK17, Ki-67, and p53 [29]. In an Indian randomized clinical trial, Krishnan et al. reported that chemiluminescence combined with toluidine blue achieved higher diagnostic accuracy for detecting dysplasia in tobacco-associated oral lesions than chemiluminescence combined with Lugol’s iodine [30]. Saini et al., in a randomized clinical trial from Italy, showed that adjunctive autofluorescence examination using OralID increased both sensitivity and specificity for detecting oral potentially malignant lesions compared with conventional white-light examination alone [31]. A Brazilian pilot clinical study by Simonato et al. reported that fluorescence visualization improved sensitivity, specificity, and overall diagnostic accuracy for detecting epithelial dysplasia and oral potentially malignant disorders relative to white-light examination [32,33]. A prospective clinical trial conducted in the United States and Australia by Swathi et al. showed that fluorescent molecular imaging more accurately identified the sentinel margin than surgeon assessment, with higher agreement with final histopathology and improved real-time detection of the closest tumor margins during oral cancer resection [34,35]. Finally, a prospective diagnostic accuracy study from the United States by Yang et al. showed that combining autofluorescence imaging with high-resolution microendoscopy improved detection accuracy for moderate dysplasia or worse compared with either modality used alone [36].

#### 3.3.1. Diagnostic Assessment Studies

A subset of the included studies evaluated adjunctive light-based technologies for chairside diagnostic assessment of oral mucosal lesions. These investigations primarily focused on autofluorescence visualization, chemiluminescence-enhanced examination, or multimodal optical imaging systems used during routine clinical assessment to support lesion triage and biopsy decision-making.

Autofluorescence-based devices were the most frequently studied modality within this domain. These studies generally assessed the ability of fluorescence visualization loss to identify epithelial dysplasia or squamous cell carcinoma, using histopathological examination as the reference standard. Across studies, autofluorescence demonstrated relatively high sensitivity for detecting moderate-to-severe dysplasia and invasive carcinoma, while specificity was more variable and frequently lower, particularly in the presence of inflammatory or benign reactive lesions. Variability in reported performance parameters was influenced by differences in lesion spectrum, threshold definitions for fluorescence loss, examiner experience, and clinical setting.

Chemiluminescence-based systems were evaluated in a smaller number of studies. These investigations typically assessed the enhancement of acetowhite changes following acetic acid rinse as a marker of epithelial abnormality. Diagnostic performance estimates were heterogeneous, with sensitivity ranging from moderate to high depending on lesion composition, and specificity generally limited by false-positive findings in hyperkeratotic or inflammatory conditions. In some studies, adjunctive staining methods were combined with chemiluminescence to improve discrimination; however, methodological variability limited direct comparison. Multimodal optical approaches, including combinations of autofluorescence imaging with high-resolution microendoscopy or molecular fluorescence imaging, were also reported. These systems aimed to improve diagnostic discrimination by integrating structural and metabolic optical signals. Preliminary findings suggested improved concordance with histopathological grading compared with single-modality visualization alone, although sample sizes were limited and thresholds for positivity were not uniformly standardized.

Across diagnostic assessment studies, substantial heterogeneity was observed with respect to unit of analysis (lesion-based versus patient-based), positivity criteria, and verification procedures. In several studies, not all clinically negative lesions underwent biopsy, introducing potential verification bias. For these reasons, quantitative pooling of sensitivity and specificity estimates was not undertaken.

#### 3.3.2. Intraoperative Optical Guidance Studies

A separate group of included studies evaluated light-based optical technologies in the intraoperative management of oral squamous cell carcinoma. These investigations did not primarily assess diagnostic test accuracy for lesion detection but rather examined the utility of fluorescence-guided techniques in surgical decision-making.

Fluorescence-guided surgery studies assessed the role of optical visualization in delineating tumor margins, identifying sentinel margins, or detecting residual malignant tissue during resection. In these trials, outcomes included concordance between fluorescence-defined margins and final histopathology, closest margin distance, local recurrence rates, and, in some cases, overall survival. While fluorescence guidance improved intraoperative visualization of tumor extent in certain settings, its impact on long-term oncologic outcomes was variable across studies. Near-infrared fluorescence imaging was also evaluated for nodal mapping and enhancement of lymph node yield during neck dissection. These studies assessed whether fluorescence guidance increased the number of harvested nodes or improved identification of metastatic involvement. The primary outcomes in this context were surgical performance metrics rather than sensitivity or specificity for mucosal dysplasia detection.

Molecular fluorescence imaging approaches were used in some trials to enhance real-time identification of tumor tissue during resection. These systems aimed to improve intraoperative discrimination between malignant and non-malignant tissue based on targeted fluorescence probes. Although promising in terms of margin detection accuracy and agreement with final pathology, these technologies remain at an early stage of clinical integration. Given the fundamentally different clinical objectives and outcome measures of intraoperative guidance studies compared with diagnostic assessment studies, results from these domains were not combined. The intraoperative evidence reflects the potential of light-based optical systems to support surgical precision rather than to function as screening or triage tools.

## 4. Discussion

### 4.1. Results in the Context of Other Evidence

The findings should be interpreted within the context of two distinct clinical applications. Diagnostic assessment studies evaluated the ability of light-based technologies to identify mucosal lesions requiring biopsy, whereas intraoperative studies assessed their utility in surgical margin delineation and nodal mapping. These applications address different clinical objectives and should not be interpreted interchangeably.

The findings of this systematic review are broadly concordant with, and extend, the existing evidence base concerning adjunctive light-based screening technologies for oral potentially malignant disorders and squamous cell carcinoma. Across modalities, the diagnostic accuracy parameters observed in the included studies mirror those reported in prior high-quality systematic reviews and meta-analyses, underscoring both the reproducibility of these technologies’ performance characteristics and the persistence of their inherent limitations. Collectively, the evidence suggests that light-based adjuncts consistently demonstrate high sensitivity for the detection of dysplasia and carcinoma, while specificity remains variable and frequently suboptimal, particularly in real-world clinical settings. Autofluorescence imaging was the most extensively investigated modality among the included studies, reflecting its widespread clinical availability and long-standing interest as a non-invasive adjunct to conventional oral examination. The pooled diagnostic performance observed in this review is in close agreement with prior meta-analyses reporting sensitivity estimates in the range of 81–90% and specificity values between 50% and 72% [37]. The 2021 Cochrane systematic review, encompassing 63 studies and nearly 8000 lesions, reported summary sensitivity and specificity estimates of 0.87 and 0.50, respectively, for light-based detection systems [38]. Similarly, Buenahora et al. reported a pooled sensitivity of 86% and specificity of 72% for autofluorescence, with an AUC of 0.86, indicating good overall discriminative ability despite limited specificity [39]. The close alignment of these estimates with those observed in the present review reinforces the robustness of these findings across diverse populations and study designs. The consistently high sensitivity of autofluorescence imaging has important clinical implications, particularly in the context of risk stratification and exclusion of high-grade disease. Several large prospective studies have demonstrated sensitivity approaching 95–100% for the detection of moderate to severe dysplasia and invasive carcinoma, accompanied by negative predictive values exceeding 98% [40,41]. These findings suggest that lesions exhibiting retention of autofluorescence are highly unlikely to harbor advanced dysplasia or malignancy. In practical terms, this characteristic supports the use of autofluorescence as a rule-out tool, potentially reducing patient anxiety and limiting unnecessary biopsies in clinically equivocal lesions that do not demonstrate fluorescence visualization loss [41]. Despite these advantages, the low specificity of autofluorescence remains a major and clinically relevant limitation. High false-positive rates are consistently reported, leading to increased numbers of biopsies, higher healthcare costs, and potential psychological burden for patients [37,42]. The underlying cause of this limited specificity lies in the non-specific nature of fluorescence loss, which may occur not only in dysplastic or malignant tissue but also in benign inflammatory, traumatic, or vascular lesions [43]. Inflammatory changes, increased epithelial thickness, altered stromal collagen, and enhanced vascularity can all reduce tissue autofluorescence, mimicking neoplastic change. Chemiluminescence-based systems demonstrated even more variable diagnostic performance across studies. Meta-analytic data indicate pooled sensitivity estimates ranging from 67% to 85%, with specificity frequently below 55% [37,39]. The American Dental Association evidence-based guideline reported particularly low specificity values, as low as 31%, when chemiluminescence was used in patients with clinically evident suspicious lesions [44]. Compared with autofluorescence, chemiluminescence appears to offer no meaningful advantage in diagnostic accuracy and may further exacerbate false-positive findings, limiting its utility as a standalone screening or diagnostic adjunct. An additional concern with chemiluminescence is its preferential detection of leukoplakic lesions, with reduced sensitivity for erythroplakia and mixed red lesions, which paradoxically carry a higher risk of malignant transformation [43,45,46]. Early clinicopathological studies demonstrated extremely low specificity, with one study reporting a specificity of only 14.2% despite 100% sensitivity [47]. While the addition of toluidine blue vital staining to chemiluminescence has been proposed as a means to improve diagnostic performance, evidence supporting combined approaches remains limited. Although combined tissue reflectance and vital staining achieved improved specificity in guideline-level evidence, these findings require confirmation in well-designed prospective trials [44]. Comparison with conventional oral examination provides essential clinical context. Conventional examination alone has demonstrated moderate sensitivity but relatively higher specificity compared with light-based adjuncts [39]. Autofluorescence consistently improves sensitivity at the expense of specificity, resulting in a diagnostic trade-off rather than a clear overall advantage [39,48]. These findings support the interpretation that light-based technologies should be viewed as adjuncts to, rather than replacements for, thorough clinical examination and histopathological confirmation. Professional guidelines and public health bodies have adopted a cautious stance regarding routine use of light-based adjuncts. The American Dental Association concluded that while adjunctive tests may assist in identifying lesions requiring biopsy, none can substitute for histopathological examination, which remains the diagnostic gold standard [44]. Similarly, the U.S. Preventive Services Task Force found insufficient evidence to recommend routine screening using adjunctive technologies in asymptomatic populations, particularly in primary care settings [49]. Emerging evidence suggests that light-based adjuncts may nevertheless have value in specific, targeted clinical applications. Autofluorescence has demonstrated utility in surgical margin delineation, often extending beyond clinically visible lesion boundaries and correlating with areas of dysplasia identified by vital staining [50,51]. Additional applications include guidance of biopsy site selection within heterogeneous lesions and longitudinal surveillance of oral potentially malignant disorders [40,41,42,43,52,53]. Recent advances in artificial intelligence and machine learning represent a promising strategy to address the specificity limitations of current technologies. Quantitative image analysis and deep learning approaches applied to autofluorescence images have achieved diagnostic accuracy exceeding 97% in experimental and early clinical studies [54,55,56,57]. These systems may enable objective, reproducible interpretation of optical signals, reducing operator dependence and false-positive rates [58,59,60,61,62,63,64]. However, most studies remain preliminary, and external validation in diverse clinical settings is required before routine clinical adoption. Advanced optical imaging modalities, including fluorescence spectroscopy, diffuse reflectance spectroscopy, and 5-aminolevulinic acid-induced protoporphyrin IX fluorescence, have demonstrated superior specificity and overall diagnostic accuracy compared with conventional autofluorescence imaging [42]. While these technologies show promise, their implementation is currently limited by cost, technical complexity, and availability, necessitating further validation and cost-effectiveness analyses.

### 4.2. Limitations of the Evidence

An important limitation of the available evidence is the substantial heterogeneity across included studies with respect to study design, patient populations, index tests, reference standards, and diagnostic thresholds. Both randomized and non-randomized designs were included, with the latter introducing an increased risk of selection and spectrum bias. Many studies were conducted in secondary or tertiary referral centers, often enrolling patients with clinically evident lesions or elevated risk profiles, which may overestimate diagnostic accuracy compared with general or primary care populations. Considerable variability was observed in the implementation of light-based technologies, including differences in device type, wavelength, illumination conditions, examiner training, and criteria used to define a positive test result. In many studies, diagnostic outcomes relied on subjective visual interpretation rather than quantitative or standardized criteria, increasing the risk of inter- and intra-observer variability. Blinding of examiners to histopathological outcomes was inconsistently reported, raising concerns regarding observer bias. Follow-up and verification procedures also varied widely. While histopathology was generally used as the reference standard, not all lesions underwent biopsy, particularly those deemed negative by adjunctive screening, introducing partial verification bias. In addition, small sample sizes were common, limiting statistical power and increasing uncertainty around sensitivity and specificity estimates. Few studies incorporated advanced optical imaging, quantitative analysis, or objective biomarkers that could have strengthened diagnostic validation. Finally, most studies assessed cross-sectional diagnostic performance rather than longitudinal outcomes, limiting conclusions regarding the ability of adjunctive technologies to predict malignant transformation over time. As a result, while high sensitivity was frequently observed, the clinical implications of false-positive and false-negative findings remain incompletely characterized.

### 4.3. Limitations of the Review

This review has several inherent limitations. Although conducted as a systematic review, quantitative synthesis through meta-analysis was not feasible for all outcomes due to pronounced heterogeneity in study designs, index tests, reference standards, and reporting of diagnostic accuracy metrics. Differences in positivity thresholds and inconsistent reporting of true positive, false positive, true negative, and false negative values further limited data pooling. Interpretation of studies using split-mouth or lesion-based designs requires caution, as clustering effects and non-independence of observations were rarely addressed statistically. Furthermore, publication bias cannot be excluded, as studies reporting favorable diagnostic performance may be more likely to be published. Despite these limitations, this review provides a comprehensive and structured synthesis of the current evidence base, highlighting consistent diagnostic patterns, methodological weaknesses, and key areas requiring further investigation. Because this review encompassed both diagnostic assessment and intraoperative guidance applications, heterogeneity in study design, outcome measures, and reference standards precluded quantitative synthesis across domains. The separation of these domains was therefore essential to maintain conceptual clarity and methodological rigor.

### 4.4. Implications

The findings of this review have several important implications for both clinical practice and future research. Adjunctive light-based screening technologies demonstrate consistently high sensitivity for detecting dysplastic and malignant lesions, supporting their potential role as complementary tools to conventional oral examination. However, their limited specificity underscores that these technologies should not be used as standalone diagnostic tests and cannot replace histopathological confirmation. Future research should prioritize well-designed, multicenter prospective diagnostic accuracy studies using standardized protocols, predefined positivity criteria, and consistent reference standards. Studies conducted in primary care and general dental settings are particularly needed to assess real-world performance and clinical utility. Integration of quantitative image analysis, spectroscopy, and artificial intelligence may help address current specificity limitations and reduce operator dependence. Longitudinal studies evaluating the predictive value of adjunctive screening for malignant transformation are also warranted. Until such evidence is available, light-based technologies should be viewed as adjuncts that may assist in lesion detection, biopsy site selection, and surveillance, rather than definitive diagnostic tools. Careful clinical judgment and histopathological assessment remain essential for accurate diagnosis and management of oral potentially malignant disorders and squamous cell carcinoma.

## 5. Conclusions

This systematic review demonstrates that adjunctive light-based screening technologies consistently exhibit high sensitivity for the detection of oral dysplastic and malignant lesions, supporting their role as complementary tools to conventional oral examination. Autofluorescence demonstrates a consistent pattern of higher sensitivity than specificity across heterogeneous study designs; however, variability in thresholds, clinical settings, and verification procedures limits definitive conclusions regarding magnitude of effect. However, the limited and variable specificity observed across studies remains a significant constraint, resulting in high false-positive rates and restricting clinical applicability as standalone screening methods. Chemiluminescence-based systems do not appear to provide meaningful diagnostic advantage over autofluorescence and are similarly limited by poor specificity. Given the substantial heterogeneity in study design, diagnostic thresholds, and clinical settings, current evidence does not support routine population-level screening using adjunctive light-based technologies alone. These modalities should be used judiciously, in conjunction with careful clinical examination and mandatory histopathological confirmation of suspicious findings. Future research should focus on standardized, multicenter diagnostic accuracy studies conducted in primary and secondary care settings, incorporation of quantitative image analysis and artificial intelligence to improve specificity, and longitudinal assessment of malignant transformation risk. Until such evidence is available, adjunctive light-based technologies should be regarded as supportive diagnostic aids rather than definitive diagnostic instruments in the management of oral potentially malignant disorders and squamous cell carcinoma. Interpretation of these findings is limited by substantial heterogeneity in device characteristics, lesion spectrum, study design, and reference standard application, as well as by the absence of formal meta-analysis. Current evidence therefore supports the use of light-based technologies as adjunctive tools rather than standalone diagnostic methods.

## Figures and Tables

**Table 1:** Selection criteria for papers included in the systematic review.

Inclusion Criteria	Exclusion Criteria
Randomized controlled trials	Narrative reviews
Non-randomized controlled trials	Systematic reviews and meta-analyses
Prospective or retrospective diagnostic accuracy studies	Letters to the editor
Full-text articles available	Conference abstracts or proceedings without full text
Human studies	Animal or in vitro studies
English-language publications	Studies evaluating therapeutic laser interventions only
Adult patients (≥18 years)	Studies without histopathological confirmation of diagnosis
Use of adjunctive light-based screening technologies	
Histopathological examination as reference standard	
Low or moderate risk of bias	

**Table 2:** Risk of Bias assessment for the included studies.

Study	Laser Parameters Reported	Protocol Clearly Described	Diagnosis Confirmed	Control/Placebo Group	Randomization Described	Statistical Analysis Appropriate	Transparent Outcome Reporting	Follow-Up & Attrition Reported	Funding/COI Disclosed
Christensen et al., 2019 [26]	Yes	Yes	No	Yes	Yes	Yes	Yes	Yes	Yes
Cicciù et al., 2017 [27]	No	No	No	No	No	Limited	Yes	No	Unclear
Durham et al., 2020 [28]	No	Yes	No	Yes	Yes	Yes	Yes	Yes	Yes
Ikeda et al., 2020 [29]	No	Yes	No	No	No	Yes	Yes	Yes	Yes
Krishnan et al., 2022 [30]	Yes	Yes	No	Yes	Yes	Yes	Yes	Yes	Yes
Saini et al., 2019 [31]	Yes	Yes	No	No	No	Yes	Yes	Limited	Yes
Simonato et al., 2017 [32]	No	Partial	No	No	No	Yes	Yes	No	Unclear
Swathi et al., 2021 [33]	Yes	Yes	No	No	No	Yes	Yes	Limited	Yes
Yang et al., 2018 [34]	No	Yes	No	Yes	No	Yes	Yes	Yes	Yes

COI—conflict of interest.

**Table 3:** A general overview of the studies.

Study	Country	Study Design	Main Outcomes
Christensen et al., 2019 [26]	Denmark	Randomized clinical trial	Near-infrared fluorescence–guided neck dissection significantly increased lymph node yield compared with conventional neck dissection (primary endpoint: lymph node yield in levels Ib–III).
Cicciù et al., 2017 [27]	Italy	Clinical study	Fluorescence imaging described as a non-invasive adjunct for early diagnosis of oral premalignant and malignant lesions; no quantitative clinical outcome defined.
Durham et al., 2020 [28]	Canada	Multicenter randomized clinical trial	Fluorescence visualization–guided surgery did not reduce 3-year local recurrence compared with conventional surgery; no significant differences in margin status, survival, or recurrence.
Ikeda et al., 2020 [29]	Japan	Prospective clinical study	Fluorescence visualization delineated oral epithelial dysplasia adjacent to early tongue squamous cell carcinoma comparably to iodine vital staining; FV loss correlated with higher expression of CK17, Ki-67, and p53.
Krishnan et al., 2022 [30]	India	Randomized clinical trial	Chemiluminescence combined with toluidine blue showed higher diagnostic accuracy than chemiluminescence with Lugol’s iodine for detecting dysplasia in tobacco-associated oral lesions.
Saini et al., 2019 [31]	Italy	Randomized clinical trial	Adjunctive autofluorescence examination with OralID increased sensitivity and specificity for detecting oral potentially malignant lesions compared with conventional white-light examination alone, supporting its value as an adjunctive screening tool.
Simonato et al., 2017 [32]	Brazil	Pilot clinical study	Fluorescence visualization increased sensitivity, specificity, and diagnostic accuracy for detecting epithelial dysplasia and oral potentially malignant disorders compared with white-light examination.
Swathi et al., 2021 [33]	USA, Australia,	Prospective clinical trial	Fluorescent molecular imaging more accurately identified the sentinel margin than surgeon assessment, showing higher agreement and correlation with final pathology, and enabling improved real-time detection of the closest tumor margins during oral cancer resection.
Yang et al., 2018 [34]	United States	Prospective diagnostic accuracy study	Combined autofluorescence imaging and high-resolution microendoscopy improved detection accuracy of moderate dysplasia or worse compared with either modality alone.

FV, fluorescence visualization; CK17, cytokeratin 17; Ki-67, Ki-67 proliferation index; p53, tumor protein p53; OralID, autofluorescence oral screening device (proprietary name).

## Data Availability

No new data were created or analyzed in this study.

## Supplementary Materials

The following supporting information can be downloaded at https://www.mdpi.com/article/10.3390/jcm15051693/s1, Supplementary Materials S1: PRISMA 2020 checklist.
